# A Computational Study of Elongation Factor G (EFG) Duplicated Genes: Diverged Nature Underlying the Innovation on the Same Structural Template

**DOI:** 10.1371/journal.pone.0022789

**Published:** 2011-08-04

**Authors:** Tõnu Margus, Maido Remm, Tanel Tenson

**Affiliations:** 1 Department of Bioinformatics, Institute of Molecular and Cell Biology at University of Tartu, Tartu, Estonia; 2 Estonian Biocentre, Tartu, Estonia; 3 Institute of Technology at University of Tartu, Tartu, Estonia; Université Paris-Sud, France

## Abstract

**Background:**

Elongation factor G (EFG) is a core translational protein that catalyzes the elongation and recycling phases of translation. A more complex picture of EFG's evolution and function than previously accepted is emerging from analyzes of heterogeneous EFG family members. Whereas the gene duplication is postulated to be a prominent factor creating functional novelty, the striking divergence between EFG paralogs can be interpreted in terms of innovation in gene function.

**Methodology/Principal Findings:**

We present a computational study of the EFG protein family to cover the role of gene duplication in the evolution of protein function. Using phylogenetic methods, genome context conservation and insertion/deletion (indel) analysis we demonstrate that the EFG gene copies form four subfamilies: EFG I, spdEFG1, spdEFG2, and EFG II. These ancient gene families differ by their indispensability, degree of divergence and number of indels. We show the distribution of EFG subfamilies and describe evidences for lateral gene transfer and recent duplications. Extended studies of the EFG II subfamily concern its diverged nature. Remarkably, EFG II appears to be a widely distributed and a much-diversified subfamily whose subdivisions correlate with phylum or class borders. The EFG II subfamily specific characteristics are low conservation of the GTPase domain, domains II and III; absence of the trGTPase specific G2 consensus motif “RGITI”; and twelve conserved positions common to the whole subfamily. The EFG II specific functional changes could be related to changes in the properties of nucleotide binding and hydrolysis and strengthened ionic interactions between EFG II and the ribosome, particularly between parts of the decoding site and loop I of domain IV.

**Conclusions/Significance:**

Our work, for the first time, comprehensively identifies and describes EFG subfamilies and improves our understanding of the function and evolution of EFG duplicated genes.

## Introduction

Gene duplication is postulated to have played an important role in prokaryotic evolution; the divergence accumulated in the sequences of new gene copies could be considered as a major contribution to the evolution of novel gene functions [1], [2], [3]. Complete genome sequences have been surveyed for trGTPases [4], [5] but present knowledge does not include systematically structured information concerning EFG duplications in bacteria.

Elongation factor G (EFG) is an indispensable protein present in bacteria (EFG), archea (aEF2), and eukaryotes (eEF2) [6]. Data gathered since the 1960s concerning EFG are mainly based on the *Escherichia coli* (*E. coli*) model system [7], [8]. EFG is the translocase of translation, it catalyzes the movement of the peptidyl-tRNA from the A-site to the P-site and deacetylated tRNA from the P-site to the E-site of the ribosome [9], [10]. In addition, EFG together with ribosome recycling factor (RRF) participates in the disassembly of the post-termination ribosomal complex [11], [12]. These EFG functions, catalyzing translocation and ribosome recycling, are indispensable to cells.

EFG belongs to the translational GTPase (trGTPase) superfamily, whose bacterial members (IF-2, EF-Tu, EFG, SelB, CysN, RF3, TypA/BipA, LepA, Tet/RPP) are associated with diverse biological roles [13], [14], [15], [16]. Four large families, for which an ancestral protein existed in the last universal common ancestor (LUCA), can be identified [17]. The members of the EFG/EF2 family (EFG, TypA/BipA, LepA, RF3, and Tet/RPP) are successful descendants of the functional diversification resulting from gene duplications.

It is believed that highly expressed genes evolve slowly and that their duplication is avoided or counter-selected, which could be related to the unique structural or functional features that constrain their sequences [18], [19]. However, data obtained from complete bacterial genomes have demonstrated that two highly expressed trGTPases genes, *tuf* (EF-Tu) and *fus* (EFG), are often represented by multiple copies [4], [20]. Moreover, EF-Tu duplicates are restricted to a few phylogenetic groups (*Proteobacteria*, *Thermus-Deinococcus* and class *Clostridia*), whereas genomes containing duplicate genes for EFG are represented among all phyla [5]. Compared with EF-Tu, where both copies are almost identical due to gene conversion between paralogues [21], the EFG gene family is significantly divergent with the paralogues sharing approximately 30–40% identity [5]. In order to investigate how selective pressures avoid or favor divergence act on EFG duplicate genes, EFG subfamilies were identified and characterized. Phylogenetic reconstruction of bacterial EFGs revealed that during the course of evolution EFG gene multiplications have evolved under differential selective pressures, resulting in four distinct subfamilies: EFG I; spdEFG1; spdEFG2; and EFG II.

Despite of the fact, that the great potential of the gene duplication as the process involved in creating biological novelty is well known, there is still not enough information concerning the mechanisms responsible for creating functional divergence. Recently, the functional divergence of different EFG gene duplicates has attracted much attention; independent studies have revealed that EFG functions vary within the EFG family. For example, Connell et al. demonstrated that EF-G-2 in *Thermus thermophilus* binds and hydrolyzes GTP and is active in poly(Phe) synthesis [22]. Seshadri et al. demonstrated that MsmEF-G-2 in *Mycobacterium smegmati*s binds guanine nucleotides but lacks ribosome-dependent GTPase activity characteristic of EFGs [23]. Another study demonstrated that translocation and ribosome recycling, two functions catalyzed by EFG, have been split between EFG paralogues in *Borrelia burgdorferi* [24]. Therefore, the EFG family provides an interesting example of the fate of a duplicated gene and could be used as a model for in-depth study of changes that arise through gene duplication and divergence.

One of the aims of this study concerning gene duplications was to detect the rearrangements in functional regions of EFG that could be involved in creating altered functions on the same structural template. A large fraction of EFG duplications that have not previously been described were investigated as a separate EFG subfamily (the EFG II subfamily). This group of EFG duplications was chosen owing to its wide distribution among all bacterial species and a high degree of divergence, which could be accompanied by functional novelty. The detailed analysis of the EFG II subfamily is essential for understanding how the duplication events contribute to evolutionary advantage.

## Results and Discussion

### Identification and characterization of EFG subfamilies

**Figure 1:**
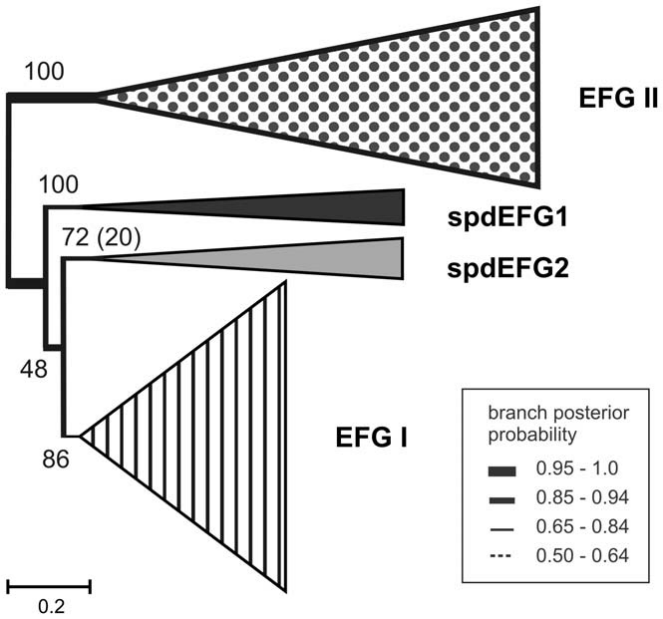
Phylogenetic tree showing four subfamilies of the EFG duplications. The EFG tree was inferred by Bayesian inference (BI) and Maximum likelihood (ML) using 214 sequences from 99 completed genomes. Sequences from the same subfamily are compressed and shown as triangles. Triangle height corresponds to evolutionary distance and triangle base corresponds to the number of compressed sequences involved. EFG subfamilies are indicated by a striped triangle (EFG I), grey triangle (spdEFG2), black triangle (spdEFG1) and filled triangle (EFG II). Support for major branches is indicated by maximum likelihood bootstrap percentage (MLBP) and is shown by numbers. Branch thickness is drawn according to BI posterior probability (BIPP) as indicated in the inset box to the right of the figure. Scale bar shows changes per position, estimated by MrBayes.

An initial set of 305 complete genomes was used to identify duplications of EFG genes. We focused on the determination of EFG subfamilies. Therefore, data from genomes with a single EFG gene were excluded from this analysis, and the first set of sequences (214 EFG sequences) was limited to the 99 genomes that exhibited multiple EFGs. Phylogenetic trees for determining EFG subfamilies were constructed using Bayesian inference (BI) and maximum likelihood (ML) methods. We show that EFG duplicate genes form within the phylogenetic tree four subfamilies: the EFG I subfamily; the spdEFG1 subfamily; the spdEFG2 subfamily; and the EFG II subfamily (Figure 1).

Two additional types of evidence, conserved insertions or deletions in sequence alignment (conserved indels) and genome context conservation confirmed that two of the EFG subfamilies were distinct groups. Firstly, the conserved genome context characterized the EFG I subfamily, the EFG coding *fus* gene being located in the *str* operon. The *str* operon of *E. coli* contains the genes for ribosomal proteins S12 (*rpsL*), S7 (*rpsG*), and elongation factors EFG (*fus*), and EF-Tu (*tuf*) [25]. The genome context conservation analysis was performed on the initial set of 305 genomes; genomes with a single EFG gene and those with multiplied EFG genes were included. Secondly, the indel analysis demonstrated that the spdEFG1 has a specific three amino acid insertion with a consensus “KDG” in the switch I region (Figure S1). This conserved insertion was used to resolve the evolutionary history of the spdEFG1 genes.

**Figure 2:**
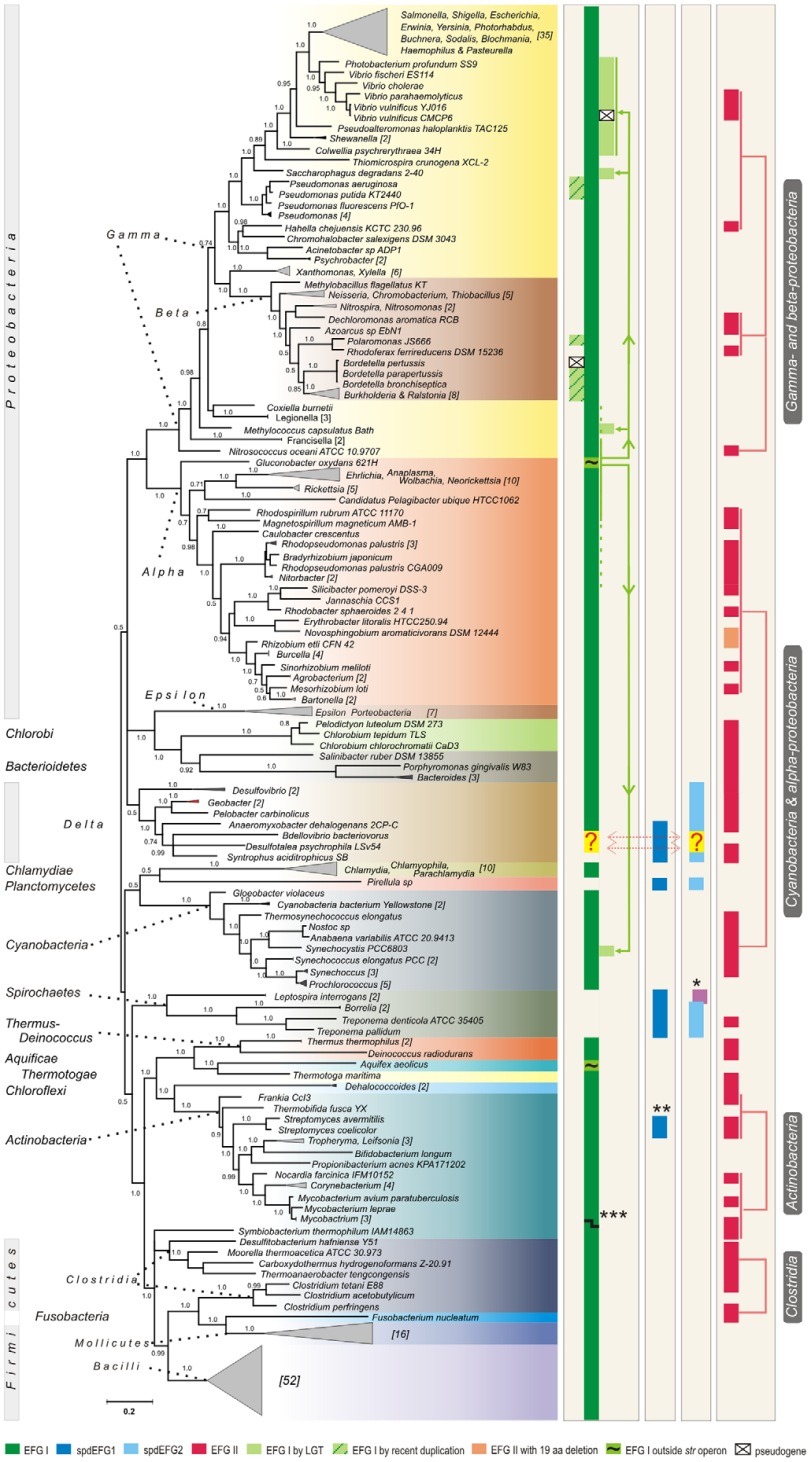
Distribution of EFG genes in bacteria and evolutionary events associated with EFG subfamilies. The color key for EFG subfamilies is as follows: green for EFG I, blue for spdEFG1, light blue for spdEFG2, and red for EFG II. Large sub-subgroups of EFG II are connected with red lines and are highlighted by white text on black. EFG I copies resulting from recent duplication(s) in *β –* and *γ – proteobacteria* are represented by diagonally striped light green boxes. Question marks and two headed arrows in *δ–proteobacteria* refer to the fact that these EFGs cannot be unambiguously determined as the members of the EFG I or spdEFG2 subfamily. The lateral gene transfer (LGT) of EFG I is represented by light green lines. Pseudogenes are represented by crossed boxes. 16S rRNA aligned sequences were retrieved from RDP II and the species tree was inferred using MrBayes. Solid triangles (in scale) indicated that sequences from more than one organism were used; the number of compressed species is shown in brackets. Exceptions are marked with asterisks: EFG's with large deletions from *Leptospira interrogans* that were not included in tree computing (*), EFGs that are classified as spdEFG1 by both phylogenetic methods but do not contain the specific insertion (KDG) in switch I (**), frameshift after switch II in *Mycobacterium bovis* (***). Scale bar shows substitutions per position estimated by MrBayes.

We have found that the majority of bacteria studied (97%) have at least one gene for EFG I (Figure 2). The EFG I tree is provided in Figure S2. We highlight that where there is a single EFG gene in a genome it belongs to the EFG I subfamily without exceptions (Figure 2). These findings are consistent with EFGs functional importance in the cell. We note that in *E. coli*, for which there are clear and experimentally well-characterized descriptions of EFG function(s), there is a single EFG gene. The EFG I gene normally resides in the *str* operon (Figure 2). Therefore, the assumption is that after gene duplication, the original copy of the *fus* gene (*fusA*), which maintains original genome context, evolves under similar constraints in all bacteria and remains stable throughout evolution. However, there are additional EFG I genes that are acquired by LGT or recent duplications and do not reside in the *str* operon (see below).

The distribution of spdEFG subfamilies (spdEFG1 and spdEFG2) is restricted to three taxonomic divisions: ***S*** *pirochaetes*, ***P*** *lanctomycetes* and ***δ*** *-proteobacteria* (Figure 2). The prefix “spd” is composed from the first letters of taxonomic divisions where these subfamilies were found [26]. The most striking feature of the spdEFG1 and spdEFG2 is their co-occurrence in the same genome if there is no gene for EFG I present in that genome (Figure 2). It has been shown previously that spdEFG1 and spdEFG2 form distinct groups with the mitochondrial EFGs mtEFG1 and mtEFG2, respectively [26]. In cells that lack EFG I (in the phyla *Spirochaetes*, *Planctomycetes*, and in various species of *δ-proteobacteria*), the essential functions of EFG I are thought to be carried out by spdEFG1 and spdEFG2. This view is consistent with the recent work of Suematsu et al. who showed that in *B. burgdorferi* the functions of bacterial EFG are split between EFG paralogues [24]. Similarly, Tsuboi et al. demonstrated that the two functions of bacterial EFG are divided between mtEFG1 and mtEFG2 in human mitochondria [27].

This is the first time that the EFG II subfamily has been characterized as a separate EFG subfamily. Some members of the EFG II subfamily have been recognized by genome annotators as “EFG-2” or “EFG-Like”, and there are two clusters of diverged EFGs (clustering threshold 50% of identity) named “EFG-Like” in Uniprot/KB. These clusters are composed of diverged *α-proteobacteria/Cyanobacteria* (UniRef50_Q55421) and *Actinomycetes* (UniRef50_O07170) sequences, which were identified as belonging to the EFG II subfamily in the present study. EFG II sequences comprise the most numerous group of EFG duplicate genes in bacteria. The data presented here demonstrate that in the EFG phylogenetic tree the EFG II subfamily forms a separate branch, which is strongly supported by the high maximum likelihood bootstrap percentage (MLBP 100) and the Bayesian inference posterior probability (BIPP 1.0) (Figure 1).

The EFG II subfamily is highly divergent in its primary sequence; only 18% of positions were conserved within the EFG II subfamily compared with 52% overall conservation within the EFG I subfamily. In contrast to other EFG subfamilies (EFG I, spdEFG1, spdEFG2), a tendency towards an increased rate of evolution (Figure 1) and a vastly increased number of indels were evident in the EFG II subfamily (Figure S1). This could explain why the EFG II gene is always accompanied by another EFG, predominantly EFG I (Figure 2).

### The emergence and distribution of EFG subfamilies

#### EFG subfamilies have emerged from ancient duplications

Well-established phylogenetic methods have demonstrated that EFG duplicate genes form four distinct subfamilies (see above). Three independent observations support the hypothesis that the four EFG subfamilies are the result of ancient duplication. Firstly, deep branches on the EFG phylogenetic tree indicate early divergence from one another (Figure 1). Secondly, the monophyly of spdEFG1 with mtEFG1 provides evidence for a common origin for these proteins [26]. Thirdly, the presence of EFG II in almost all phyla (Figure 2) suggests that the duplication event that gave rise to the EFG II subfamily occurred early in prokaryotic evolution. As the branching order of EFG subfamilies is not unambiguously determined, it complicates the picture of how EFG subfamilies emerged. Therefore, it would be intriguing to question how many gene duplications directly gave rise to those ancient subfamilies, and at which evolutionary stage they apparently took place. However, determining which is the most ancient subfamily of EFG gene duplication(s), and their exact branching order relating to that family, remains outside the scope of current research.

#### Recent duplications and LGT in EFG subfamilies

Using current data of complete genome sequences we analyzed how recent duplications and cases of lateral gene transfer (LGT) contribute to EFG subfamilies. Interestingly, recent duplications and LGT between phyla/classes that gave rise to an additional gene have shaped the EFG I subfamily but not the EFG II subfamily (Figure 2). The occurrence of an EFG I type EFG gene outside the *str* operon in class *γ-proteobacteria* indicates a successful fixation of sequence(s) acquired laterally, although not all species from *γ-proteobacteria* share this extra EFG copy (Figure 2). Another single LGT case was detected in *Cyanobacteria* (Figure 2 and Figure S2). Unfortunately, the role of LGT in spdEFGs could not be resolved owing to the limited number of complete genomes with spdEFG coding genes. The phylogenetic analysis demonstrates that within the EFG I subfamily there is a small fraction of recent duplications (Figure 2 and Figure S2). Recent duplications were identified as the source of the second EFG gene in thirteen genomes (eleven in *β-proteobacteria* and two in *γ-proteobacteria* (family *Pseudomonas*) Figure S2). The high identity of EFG I gene copies at protein level indicates retention of original function but does not supply us with sufficient information to discuss about duplicates fate.

#### Predicting fate of recent duplicates

In order to investigate how our data will fit with gene duplicate retention models we used the model derived from data of small-scale gene duplications [28]. Input for these models are values of d_S_ (substitutions per synonymous site) and d_N_ (substitutions per non-synonymous site) calculated as a cumulative value for the pair of sequences by using PAL2NAL [29]. The figure of d_N_ as the function of d_S_ was reproduced by using equations (4) and (5) [28] where our data points were added (Figure S3). All data points exceed lower quintile of 90% confidence interval of neo-functionalization model for mammals. When consider bigger population size and shorter generation time, specific for bacteria (data points will shift close to mean trend-line), our data fit with mammals neo-functionalization model even better (Figure S3). The same models gene death rate function (Weibulll survival function) predicts that 95% of gene duplicates have lost before gene copy starts evolve under purifying selection [28]. To find most parsimonious place of gene duplication event on species tree for recent duplicates (in *β- and γ-proteobacteria*) a reconciliation tree between gene tree (EFG I) and species tree was computed by SoftParsMap [30]. Two alternative scenarios of gene duplications are mapped into improved species tree (Figure S4). The first scenario, one duplication/ten deletions, leads to situation where 86% of genomes have lost a duplicate and therefore supports neo-functionalization model (Weibulll survival function predicts 95% losses). The second scenario, two duplication/four deletions, reveals that only 16% of genomes have lost a duplicate (versus predicted 95%) and, therefore contradicts with the neo-functionalization model but supports gene dosage model. Moreover, high identity at protein level between paralogues is in agreement with the increased dosage model of gene duplicate retention what postulates increasing expression from a gene that is already highly expressed with little mutational capacity [31]. However, as far as precise position of gene duplication remains ambiguous and the only parameters we estimate are cumulative values of d_N_ and d_S_, the prediction of the fate of recent duplicates can not be more precise.

It is likely that each of the four subfamilies has taken a different evolutionary route to functional diversification. Overall conservation of EFG I together with the widespread appearance of EFG II in bacteria suggests that the presence of both in the genome is the best evolutionary scenario for the majority of bacteria with duplicate EFG genes, in the light of compromise between conservation and innovation. EFG I is considered to be indispensable; any other subfamily alone cannot replace the core function performed by EFG I. However, a pair of spdEFGs can replace EFG I due to the split of EFG I functions between the paralogues (spdEFG1 and spdEFG2) [24]. Therefore, it is very probable that the function(s) that the spdEFG1 and the spdEFG2 perform is not as unique as the function(s) of the EFG II. In addition, the spdEFGs have not been distributed throughout bacteria as successfully as EFG I and the EFG II (Figure 2). The wide distribution of the EFG II subfamily evident today is likely to be an indication of the important role for this type of EFG duplication in the evolution of bacteria.

### EFG II phylogeny reveals specific sub-subgroups supported by indels

**Figure 3:**
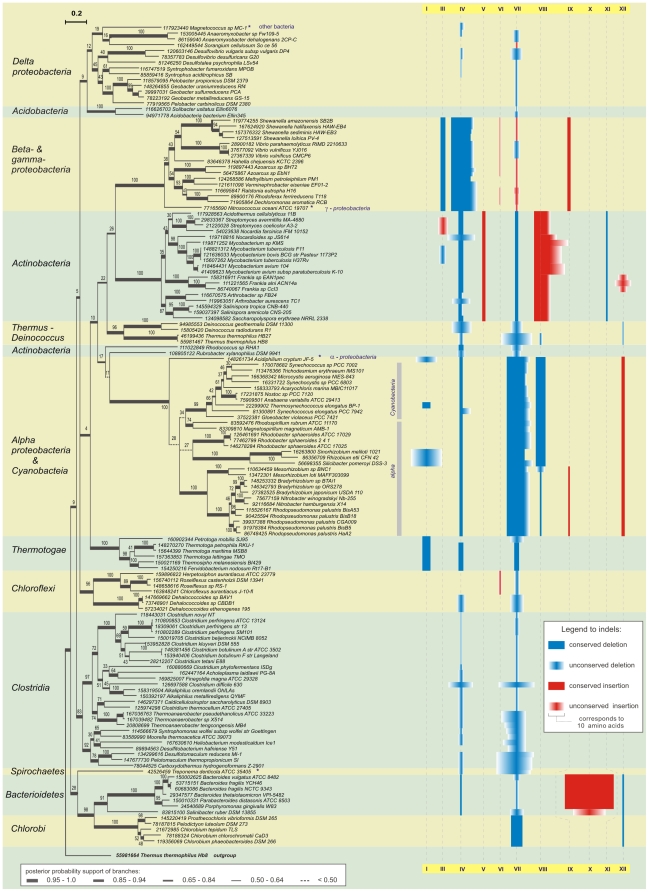
The evolutionary history of the EFG II subfamily. The evolutionary history of 141 sequences of EFG II was calculated using Bayesian inference (MrBayes) and maximum likelihood (RAxML). Bayesian inference posterior probabilities (BIPP) are indicated by line thickness and Maximum likelihood bootstrap percents (MLBP) are indicated above the nodes. EFG II sub-subgroup names are based on phyla and class names and are shown to the left of the figure. Note that *alpha-proteobacteria*/*Cyanobacteria* sub-subgroup contains sequences from two different phyla. Common indel positions are shown in blue (deletions) and in red (insertions) at the right of the figure. Conserved indels are indicated by uniformly filled rectangular boxes, indels with variable size are shown with gradient fill. Roman numerals I to XI denote the indels regions in EFG II (see also Figures 4 and S1). Scale bar shows changes per position, estimated by MrBayes.

BI and ML methods were utilized to reconstruct the phylogeny of 141 EFG II protein sequences, gathered from 590 genomes. The EFG II phylogeny is intriguing in two respects. First, relatively long branches, which are characteristic of the EFG II tree, refer to the high evolutionary speed of this gene family (Figure 3). Second, the phylogenetic signal on deeper nodes (phyla/class level) is erased. In addition, the deeper branching order is not supported by independent data as insertions/deletions (indels) (Figure 3).

**Figure 4:**
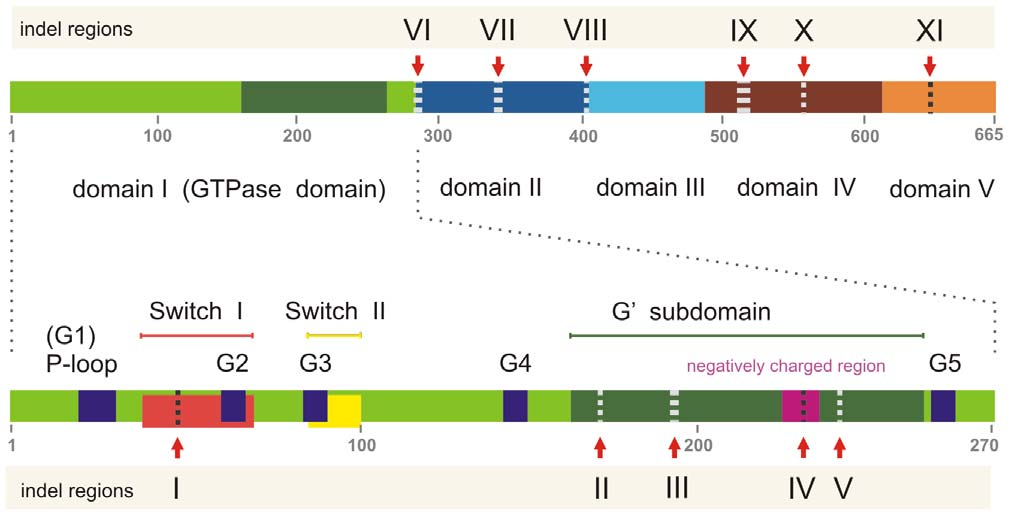
Schematic representation of EFG domain arrangement and EFG II-specific indel regions. EFG domain structure is represented by the rectangle, domains being colored as follows: green – domain I, blue – domain II, cyan – domain III, ruby – domain IV, and orange – domain V. The bottom rectangle represents the first domain (GTPase domain) in detail, the conserved motifs G1 – G5 are colored in dark blue, switch I in red, switch II in yellow, G′ motif in olive green and the negatively charged region in the G′ motif is colored magenta. Indel regions in EFG II analyzed in this our study are labeled by Roman numerals I–XI.

Indels are considered to be rare genomic changes that are more stable and easier to interpret than point mutations. Alignment regions with gaps were designated as indel regions when the specific insertion or deletion was detected in five or more sequences. Each indel region was labeled by Roman numerals from I to XI (Figure 3, Figure 4 and Figure S1). Interestingly, indels were prevalent in the EFG II subfamily but uncommon in other EFG subfamilies. Insertions and deletions in EFG II were interpreted as independent data that support the EFG II phylogeny. In addition, the indels could be regions of interest for studying functional changes in EFG II. Generally, two types of indels can be distinguished within the EFG II subfamily: (1) indels with conserved length and/or composition common to groups of closely related sequences, or (2) regions where majority of EFG II sequences have indels. One of the two indel regions within EFG II, where most sequences have indels, is region III, which is located in the G′ subdomain. The second indel-rich position in EFG II is region VI between domains I and II. Both regions predominantly contain deletions, but in *β– & γ–proteobacteria* there is a non-specific insertion in indel region VI (Figure 3). The number of indels is directly related to distance from the root of the tree. In particular, more distant group of closely related sequences (*α-proeobacteria/Cyanobacteria*, *Actionobacteria*, *β– & γ– proteobacteria*) are highly diverged and possess a large number of indels; groups near the root of the tree (*δ-proteobacteria*, *Clostridia*) are less diverged (Figure 3). However, no conserved indels were common to two different groups of closely related sequences. Therefore, it is not possible to use indels to resolve the deep branching order.

On the EFG II phylogenetic tree, sequences from the same phyla/class form monophyletic groups with one exception (see below) (Figure 3). The structure of the EFG II phylogenetic tree reveals clearly distinguishable separate groups, sub-subgroups, among the EFG II subfamily (Figure 3). These sub-subgroups are identified by the phylogenetic methods used (BI and ML) and by independent data as conserved indels (Figure 3). Phyla/class names are used to designate the sub-subgroups. Generally, the borders of the sub-subgroups correlate with phyla/class borders; no sequences from another phylum contaminate the sub-subgroups. The one exception is the case when EFG II sequences from two different phyla (*α-proteobacteria* and *Cyanobacteria*) formed one sub-subgroup (Figure 3). The common origin of the EFG II sequences forming this sub-subgroup is well supported by both tree constructing methods (BIPP 1.0, MLBP 100), and by shared deletions in regions III and VI, and insertion in region XI (Figure 3).

No LGT was observed between the sub-subgroups i.e. EFG II is not transferred between sub-subgroups. It is probable that some sub-subgroup-specific constraints could exist that avoid transfer between sub-subgroups. However, EFG II gene transfer by LGT is evident within sub-subgroups. We found an LGT case inside the *α-proteobacteria/Cyanobacteria* sub-subgroup; the donor originating from *Cyanobacteria* has been transferred to a fraction of *α-proteobacteria*. This LGT case is supported by two indels, the six amino acid deletion in region VII, and insertion in region VIII (Figure 3). In addition, in a few cases the incongruence between the 16S rRNA tree and EFG II tree could be interpreted as LGT within sub-subgroups (two cases in *β-proteobacteria* and two cases in *Actinobacteria*) (Figure S5).

### Comparison of the EFG I and EFG II subfamily

To reveal the characteristics peculiar to EFG II the variations in its primary sequence were analyzed by comparing domains and consensus elements in EFG I and EFG II. Here a short overview of EFG structural domains and assigned functions is presented.

EFG consists of five structurally well defined domains [32], [33] (Figure 4). The first domain (GTPase domain) binds and hydrolyzes GTP and is common to all P-loop GTPases. Domains III, IV and V mimic aatRNA when it is bound to EF-Tu*GTP in the ternary complex [34]. Domain III affects GTP hydrolysis and translocation [35], and domains IV and V are required for translocation but not for GTP hydrolysis [36], [37]. Translocation and ribosome dissociation into subunits at the end of translation, both functions of EFG, are GTP dependent [8], [38]. The GTPase domain (domain I) contains five consensus elements – G1, G2, G3, G4, and G5 – which form the GTP binding pocket [39], [40] (Figure 4). The overall architecture of the GTPase domain is the same in all P-loop GTPases. The translational GTPases have family specific consensus RGITI in G2 [39]. Between G4 and G5 there is an insertion with an approximate length of 90–120 aa, called the G′ subdomain [32], [41].

#### Domain conservation comparison between EFG I and EFG II

**Figure 5:**
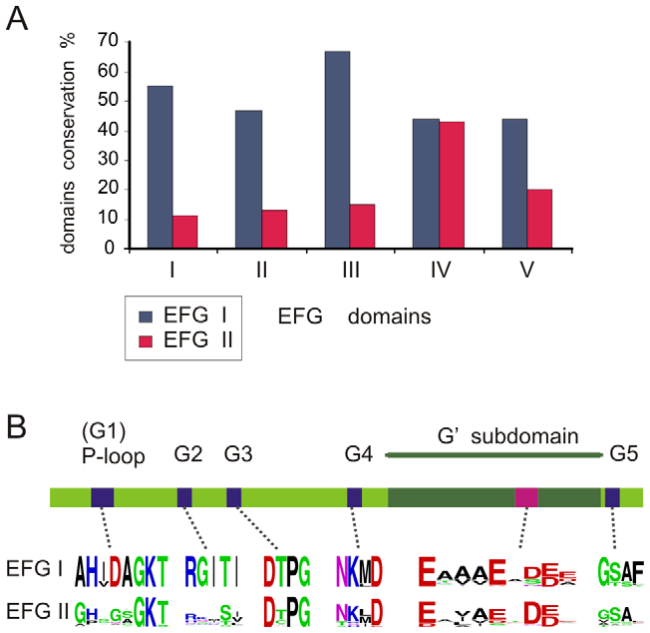
Domain and motif conservation comparisons between EFG I and EFG II. (A) Domain conservation comparison. The dark blue and red columns indicate the domain conservations of EFG I and EFG II, respectively. The domain conservation was estimated using sequence logos. (B) Motif conservation comparison. GTPase domain is indicated as a linear diagram, consensus motifs G1–G5 are shown in blue and the negatively charged region in the G′ subdomain is shown in magenta. The conservation of motifs is shown with corresponding sequence logos below their respective motifs on the linear diagram.

Domain conservation comparison between the EFG I and EFG II subfamilies revealed major differences in the first three domains (domains I, II and III) that affect GTP binding and hydrolysis. These domains are unequally conserved between EFG I and EFG II, whereas domain IV was equally conserved in both subfamilies. The conservation of domains I, II and III domains was 55%, 47% and 67%, and 11%, 13% and 15% in EFG I and EFG II respectively (Figure 5A). In addition, the relatively short domain V was found less conserved in EFG II. The high divergence of the EFG II subfamily is, therefore, predominantly related to the first three domains. Therefore, the first three domains in these subfamilies are evolving under different constraints, resulting in divergence within EFG II and homogeneity in the EFG I subfamily.

To exclude the possibility that the observed high divergence within the first three domains is caused by sub-subgroup-specific conservation of these domains, domain conservation analysis for sub-subgroups containing at least 20 sequences was carried out (*Clostridia* and *α-proteobacteria & Cyanobacteria*). The overall domain conservation was higher, and differences between domain conservations were smaller, among sub-subgroups. Furthermore, the EFG II subfamily-specific divergence of the first three domains was confirmed at the sub-subgroup level (Figure S6).

#### Motif conservation comparison between EFG I and EFG II

The GTPase domain consensus elements G1 (GhxxxGKT), G3 (DxPG), G4 (NKxD) and G5 (gSAx) were conserved in the EFG II subfamily. Moreover, the negatively charged region in the G′ subdomain, which interacts with the L7/L12 stalk on the ribosome and is crucial for inducing GTP hydrolysis [42], [43], [44], is also conserved (Figure 5). Intriguingly, the trGTPase-specific consensus RGITI in the G2 motif is relaxed in the EFG II subfamily. The redundant consensus in EFG II in the G2 motif is xxxSx. RGITI contains specific Thr, which coordinates the Mg^2+^ ion of the GTPase-bound guanine nucleotide [40]. In EFG II, Ser instead of Thr was conserved in the fourth position in the G2 motif. However, Ser instead of Thr has been observed in several P-loop GTPases (SelB – *A. aeolicus*; aIF-2-g - *M. jannaschii*; and the kinesin-myosin family) [17]. Therefore, it is concluded that the crucial position in the G2 motif (Thr/Ser), which is part of the universal ‘spring loaded’ switch mechanism for G proteins [45], is maintained.

**Figure 6:**
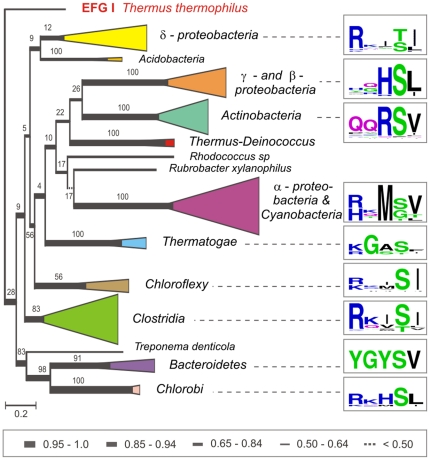
The sub-subgroups of EFG II including the G2 motif patterns. The phylogenetic tree shown in figure 3 was used and sequences of the same sub-subgroups were compressed and are shown as triangles. Triangle height corresponds to evolutionary distance and triangle base corresponds to the number of compressed sequences involved. The sequence logos at the right of the figure represent the nine EFG II sub-subgroup-specific G2 motif patterns. The broken lines connect the G2 motif patterns with the corresponding EFG II sub-subgroups. EFG I from *Thermus thermophilus* was used as an outg-roup. Scale bar shows changes per position, estimated by MrBayes.

To determine if the G2 motif conservation is maintained among closely related EFG II sequences the G2 motif variants of the EFG II sub-subgroups were analyzed. The EFG II sub-subgroup-specific G2 motif variants are as follows: RxxT/SI (*d-proteobacteria*), xxHSL (g- and *b-proteobacteria*), qqRSV (*Actinobacteria*), R/HxMS/GV (*a-proteobacteria* and *Cyanobacteria*), r/kGxSx (*Thermatogae*), r/kxxSI (*Chloroflexi*), RxxSI (*Clostridia*), YGYSV (*Bacterioidetes*), and rxhSl (*Chlorobi*) (Figure 6). Overall divergence in the G2 motif of EFG II is associated with two types of changes: (a) trGTPase-specific consensus RGITI is changed to the sub-subgroup-specific G2 motif variant and (b) Thr is replaced with Ser or exceptionally, with Gly (Figure 6).

#### Conserved and relaxed regions on the surface of EFG

The relative site-specific substitution rates for EFG subfamilies were calculated by using Rate4Site [46] and ConSurf web server [47]. One of the advantages of ConSurf in comparison to other methods is the accurate computation of the evolutionary rate by using either an empirical Bayesian method or a maximum likelihood (ML) method [48]. Thus, they can correctly discriminate between conservation due to short evolutionary time and genuine sequence conservation.

**Figure 7:**
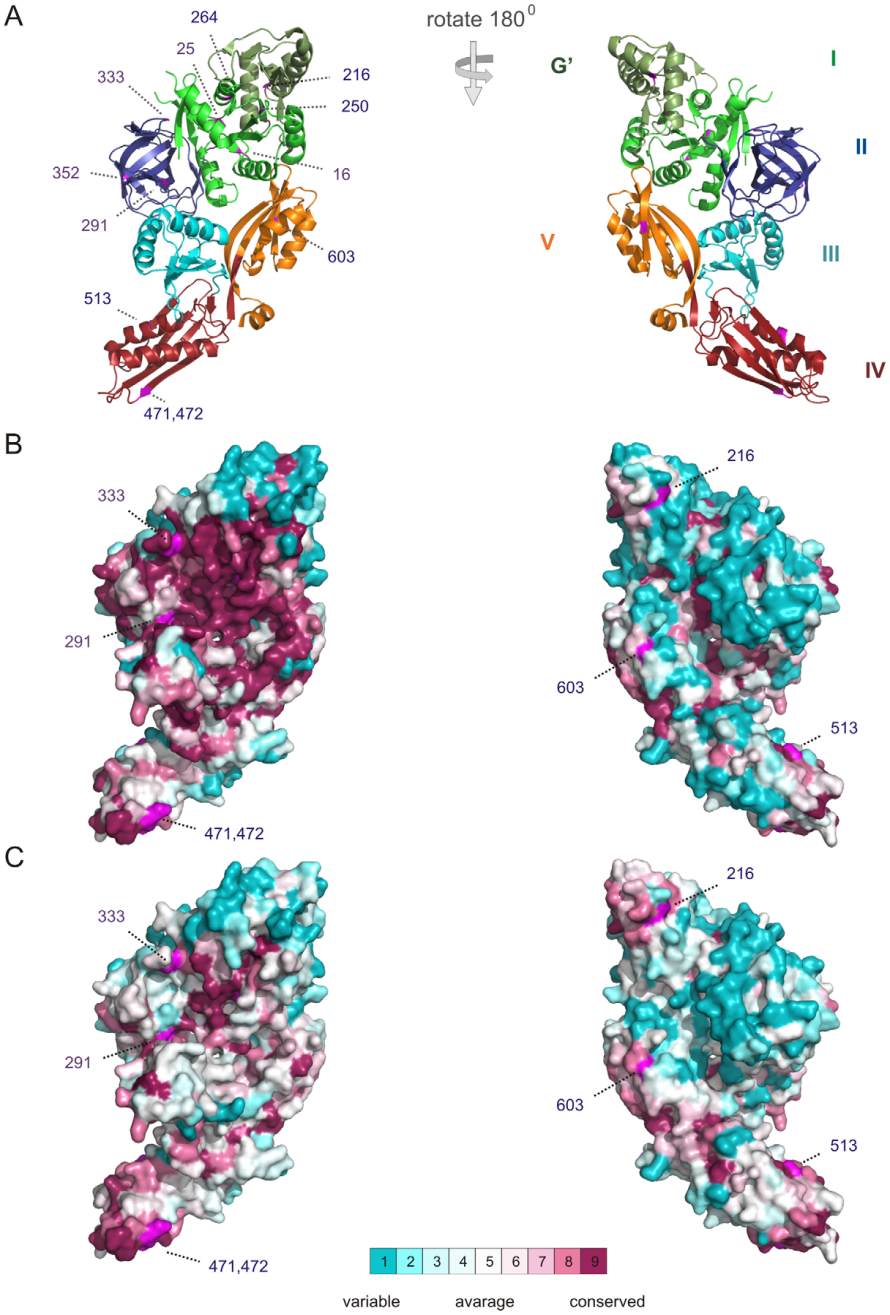
The ConSurf analysis using Bayesian Inference method for the EFG I and EFG II subfamilies. (A) The EFG crystal structure is shown as ribbon colored by domain: domains I, II, III, IV, and V are colored green (with the G′ olive green), blue, cyan, ruby and orange, respectively. Numbers are indicating type I and type II conserved positions (see below). The *left* column shows the ribosome side of the EFG; *right* column showing the opposite side following a 180° rotation about the *y*-axis. All figures in one column have the same orientation. The amino acids are colored by their conservation grades using the color-coding bar. (B) Consurf analysis of the EFG I subfamily (190 protein sequences) and (C) the EFG II subfamily (141 protein sequences) is mapped onto crystal structure surface. The run was carried out using PDB code 1FNM and the figure was generated using the PyMol script output by ConSurf.

ConSurf analysis results of the EFG I subfamily and the EFG II subfamily are mapped onto surface of the crystal structure (Figure 7 B and C respectively). The analysis reveals the high conservation of ribosome side surface of EFG I when the same region is relaxed on EFG II (Figure 7 *left* B and C respectively). Whereas, opposite sides are equally highly variable in both subfamilies (Figure 7 *right* B and C respectively). There are two regions, the tip of G′ domain and the tip of IV domain, which show moderately higher conservation in the EFG II than in the EFG I subfamily (Figure 7 *right* B and C respectively). Generally, ConSurf analysis correlates well with the domain conservation comparison results (see above) and complements to found relaxation of the first three domains of EFG II by localizing subfamily specific relaxation to ribosome side surface of EFG.

#### Conserved positions in the EFG II subfamily

Comparison of the conserved positions in EFG II (127 positions) with the conserved positions in EFG I (360 positions) revealed that the former are a subset of the latter, with a few exceptions (Figure S7). Those exceptions fall into two categories. The first category consists of the five positions where different amino acids are conserved in the EFG I and EFG II subfamilies (type I conserved positions). The second category consists of seven positions that are relaxed in EFG I but are under stronger selection in the EFG II subfamily (type II conserved positions).

Each of the five type I conserved positions is associated with substantial changes in physical-chemical properties (Table 1). The location of these five positions is restricted to the first two domains, the GTPase domain and domain II. The first two positions, 16 and 25, are in the P-loop (numbering is given according to *T. thermophilus* EFG-2 structure 1WDT). The conserved Gly16 (Ala in EFG I) increases hydrophilicity, and Leu 25 (Gly in EFG I) increases hydrophobicity (Table 1). The other three type I conserved positions (Thr-291, The Lys-352, and Gly-333) were in domain II (Table 1) and increase hydrophilicity. Seven type II conserved positions were identified in the EFG II subfamily (Table 2). Type II conserved positions are more uniformly distributed over EFG than type I conserved positions: three are located in the GTPase domain, three in domain IV and one in domain V (Table 2). Type II conserved positions are not related to considerable changes in physical-chemical properties.

**Table 1:** Type I conserved positions.

position^1^	EFG I			EFG II			location
	amino acid	hp index^2^	cons %^3^	amino acid	hp index^2^	cons %^3^	
16 (19)	Ala	1.8	100	Gly	−0.4	86	Domain I (GTPase domain)
25 (28)	Thr	−0.7	95	Leu	3.8	99	
61 (64)	Thr	−0.7	100	Ser	−0.8	76	
291 (316)	Ile	4.5	87*	Thr	−0.7	81	Domain II
333 (360)	Ala	1.8	80	Gly	−0.4	98	
352 (379)	Gly	−0.4	100	Lys (Arg)	−3.9	86 (14)**	

1 Amino acid positions are numbered according to *T. thermophilus* EFG-2 structure 1WDT. An alternative numeration (EFG-1 of *T. thermophilus*) is given in brackets.

2 Hydropathy index (positive value indicates hydrophobicity and negative value indicates hydrophilicity) [71].

3 Amino acid conservation in is given in %.

*Substitutions of Ile with Val or Leu results in minimal change in hydrophobicity.

**Lys replacement by Arg retains positive charge in this position.

**Table 2:** Type II conserved positions.

position^1^	EFG I		EFG II		differece^4^ %	location
	amino^2^ acids	%^3^	amino acid	%		
216 (224)	D, S, n *	61, 26, 10	D	88	27	Domain I (GTPase domain)
250 (258)	V, M, a	42, 33, 10	V	90	48	
264 (272)	L, M, V	38, 36, 19	L	88	50	
471 (498)	V, K, I	45, 35, 15	K	84	39	Domain IV
472 (499)	K, R, h	56, 43, 1	K	89	33	
513 (543)	E, D, n	36, 35, 6	E	87	51	
603 (633)	G, a, d	71, 9, 9	G	96	25	Domain V

1 Amino acid positions are numbered according to *T. thermophilus* EFG-2 structure 1WDT. An alternative numeration (EFG-1 of *T. thermophilus*) is given in brackets.

2 Three most represented amino acids, in a single letter code separated by commas. Amino acid shown with small letter when the conservation is <10%.

3 Percentage of conservation corresponding to the amino acids found in these positions.

4 Only those positions are shown where the difference in conservation of the most conserved amino acid exceeds 25% between the EFG I and EFG II subfamilies.

### EFG II specific conserved positions point to changed functionality

To investigate how rearrangements in functional regions could influence the capability of EFG II to perform the translocase function, a set of positions, which could be associated with altered functionality was analyzed. EFG II specific conserved positions (five type I and seven type II conserved positions) fall within the functionally important regions in the GTPase domain (domain I) and domains II, IV and V. To avoid limiting the effect of these changes within the EFG II primary sequence, these positions were mapped on to the tertiary structure of EFG (1WDT) and on to the structure of EFG with the ribosome in the pseudo-posttranslocational state [22] and posttranslocational state [49].

#### Type I conserved positions have an effect on the GTPase domain and domain II

**Figure 8:**
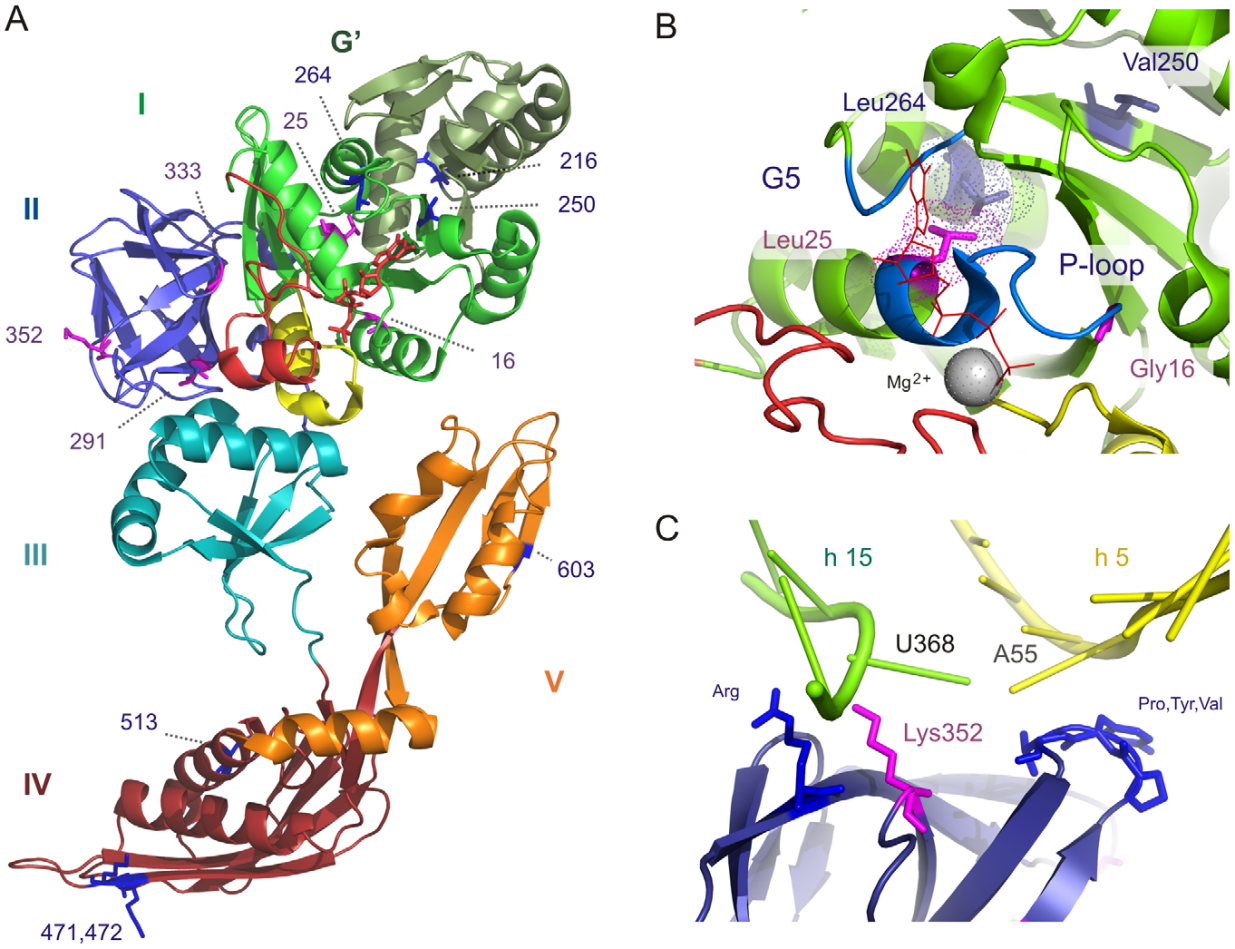
The EFG structure and location of type I and type II conserved positions. (A) The EFG structure (1WDT) is shown as ribbon colored by domain: domains I, II, III, IV, and V are colored green (with the G′ olive green), blue, cyan, ruby and orange, respectively. Switch I is red and switch II is yellow. The bound GTP is red and Mg^2+^ is shown as a grey ball. Location of type I - (violet) and type II (blue) conserved positions are shown in the structure. (B) Type I – and type II conserved positions in the GTPase domain. The Leu25 (violet stick) and its conserved interaction partner Leu264 (blue stick) from a conserved hydrophobic contact. P-loop and G5 motif are colored blue. (c) Lys352 (violet stick) is in close proximity to 16S rRNA helix 15 uridines 367 and 368. Panels a, and b are based on the EFG II structure of *Thermus thermophilus* 1WDT. For panel c the structure 2OM7 we used, which was obtained from EM studies and EFG fitting by Connelle et al. [22].

Positions 16 (Ala/Gly in EFG I/II respectively) and 25 (Thr/Leu) in the P-loop (Table 1) are located in the GTPase domain (Figure 8B). The GTPase domain binds and hydrolyzes GTP [45]. This is associated with the binding of EFG to the ribosome and translocation [50], [51], and dissociating the post-termination complex [38]. Three differentially conserved positions are located in domain II. These positions are 291 (Ile/Thr in EFG I/II respectively), 333 (Ala/Gly) and 352 (Gly/[Lys,Arg]) (Table 1 and Figure 8A). Domain II contacts the 30S subunit but no certain function has been assigned to this domain. It has been shown that domain II interacts with EFG domains I and III and with the 16S ribosomal RNA helixes 5 and 15 (h5 and h15) [22], [49].

Position 25 (the last position in P-loop) contains a well-conserved (99%) Leu that increases hydrophobicity. In the 1WDT structure, the Leu25 is located close to helix E_1_ and the G5 motif. Next to the G5 motif (7 amino acids towards the C terminus) another EFG II-specific conserved hydrophobic amino acid, Leu264, was identified (Figure S7). In the crystal structure (1WDT) the van-der-Waals radii of these two amino acids (Leu25 and Leu264) are in contact (Figure 8B), which is an indication of hydrophobic interaction between them. Moreover, the results demonstrate that Leu264 (the interaction partner of Leu25) is highly conserved (83%) in EFG II but not in EFG I (Table 2). These observations support the presence of EFG II-specific hydrophobic interactions inside the GTPase core domain, which strengthens the interaction between the P-loop and the G5 motif. This interaction increases the tightness of the GTPase core-domain and also, decreases the flexibility of the P-loop.

The crystal structures do not reveal any interactions between positions 16, 25 (Gly16 and Leu25 in EFG II) and the bound nucleotide. For position 16 it has been demonstrated that replacing Ala with Gly in aEF-2 of *Sulfolobus solfataricus* increases intrinsic GTP hydrolysis (measured in the absence of ribosomes) and decreases the Poly(Phe) synthesis rate [52]. More importantly, Connell et al. showed that EFG2 (EFG II) of *T. thermophilus* has higher intrinsic GTPase activity and a slightly lower poly(Phe) synthesis rate in cell-free assays compared with EFG-1 (EFG I) [22]. On the basis of the data, we propose that the conservation of amino acid Gly in position 16 (conserved 86%) is related to higher intrinsic GTPase activity in EFG II. Position 25, which is 99% conserved in EFG II, is likely to have the potential to modulate GTPase activity.

Domain II has not been studied extensively, and there is no specific function assigned to this domain, making it difficult to propose functional roles for the differentially conserved positions (type I conserved positions) located in this domain (positions 291, 333 and 352) (Figure 8A). In EFG II, Gly in position 333 (Ala in EFG I), which is located in the loop between beta sheets 8_2_ and 9_2_ facing towards switch I of the GTPase domain, increases hydrophilicity, which could influence the interaction between switch I and domain II. The Lys in position 352 increases the positive charge in the proximal tip of β sheet 7_2_ and contributes to an interaction with the backbone of conserved uridines U367 and U368 on the 16S rRNA helix 15 (h15) (Figure 8C) [22]. The interaction between h15/h5 and domain II of EFG is also detected on the structure (2WRK) where the ribosome is trapped with EFG in the posttranslocational state [49]. The same proximity between the β-barrel domain II and h15/h5 presents in the ribosomes in pre-translocational intermediate state (TI^PRE^) [53]. Therefore, Lys352 has the potential to influence the interaction between EFG II and the ribosome throughout different states of translocation. Two aspects are highlighted that are related to these three type I conserved amino acids located in domain II. First, all three amino acid changes increase hydrophilicity (Table 1); second, each of these three amino acids points towards different interaction partners of domain II (Figure 8B).

#### Type II conserved positions and translocation

Whereas type I conserved positions were identified in the first two domains, the type II conserved positions were located in domains I, IV and V. Asp216, Val250, and Leu264 are the three type II conserved positions located in the GTPase domain (Table 2 and Figure 8A). Val250 is turned towards the N-terminal part of the G′ subdomain, but owing to low conservation of the closest hydrophobic amino acids in the G′ subdomain no specific interactions were identified. However, considering that Val250 and Leu264 surround the G5 motif, they are probably related to modified properties of nucleotide binding center.

**Figure 9:**
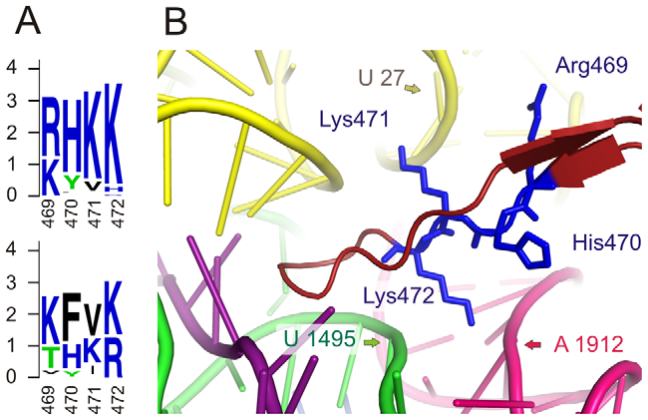
Placement of loop 1 of EFG domain IV in the ribosome decoding centre. (A) Sequence logos of conserved region of loop 1 (upper EFG II, lower EFG I). Values on y-axis correspond to bit score and x-axis shows positions according to numeration in the structure 1WDT. (B) Ribosome decoding center in post-translocational state. Colors correspond to the following components: tRNA – yellow, mRNA – violet, 16S RNA – green, 23S rRNA hot-pink, loop 1 of domain IV – brick-red. The amino acid residues of the loop I positions 469–472 were modified according to the conserved amino acids in EFG II and are shown as blue sticks. The figure is based on structure 2WRL, 2WRK [49], numeration corresponding to the structure 1WDT [22].

Two of the type II conserved positions (471,472) are located in domain IV, which is required for translocation [36]. These two conserved Lys residues increase the positive charge of the loop I region (Table 2). More intriguingly, two additional adjacent positions, 469 and 470, contribute to the positive charge of that region (Figure 9A). These positions do not correspond to the threshold for single position conservation and therefore they are not shown in table 2. However, they form one single positively charged motif/region, which consists of four consecutive positions. To illustrate its interaction with the negatively charged backbone of rRNA and tRNA amino acid residues of loop I were modified *in silico* to those conserved in EFG II (Figure 9). It has previously been shown that replacing Lys with hydrophobic Ile in position 496 reduces the poly(Phe) synthesis efficiency more than twofold [54]. Therefore, it is assumed that the translation efficiency depends on the strength of the interaction between EFG and the decoding center and this interaction could increase translocation efficiency, particularly in those physiological conditions where the stronger interaction could be critical.

The divergent nature of the EFG II subfamily encourages us to ask what function(s) does this protein perform really? On the one hand, in the case of the EFG II subfamily, the weakened selection of duplicated genes can be observed as a vastly increased evolutionary speed and an increased number of indels. On the other hand, among members of EFG II subfamily there is particularly intense selection for certain characteristics, such as some positions, that are conserved throughout the entire subfamily. The presence of conserved characteristics in the otherwise highly diverged sequences of EFG II, which appear to correlate with unique functional peculiarities, can guide and inform the design of future experiments in this area of research. Our results suggest that EFG II specializes in some roles assigned to EFG I, but the possibility of functional shift should be also considered. The positions that are differentially conserved in EFG I and in EFG II (type I conserved positions), and the positions under stronger selection in EFG II (type II conserved positions) are the specific characteristics that provide information about functional divergence. They pinpoint the set of specific characteristics that open the door to further biochemical studies targeting the EFG's altered functionality.

## Materials and Methods

### Identifying EFG sequences

EFG protein sequences have been identified using HMMSEARCH [55] and TBLASTN [56] according to the procedure described by Margus et al. 2007 [5]. Searches were performed against the NCBI Ref-Seq database of completed bacterial genomes retrieved from NCBI. Three sets of EFG sequence data were used: the first contained 214 EFG sequences from 99 genomes with multiple *fus* genes; the second dataset contained EFG I sequences from genomes with single and multiple *fus* genes; the third dataset contained 141 EFG II sequences collected from 590 genomes. The first two sets were based on the Ref-Seq database of completed genomes, dated October 2006 and the third set is based on the Ref-Seq database as it was on March 2008 [57].

### Computing multiple sequence alignments

The preliminary alignment of the first dataset was carried out with MAFFT version 5.861 [58] using strategy L-INS-I. Two highly diverged EFGs from *Leptospira interrogans* were excluded from the dataset used for tree building because of extensive deletions within the sequence. The final alignment was computed with T-COFFEE [59] where, in addition to default methods, results of threading to EFG tertiary structure 1FNM with FUGUE [60] were taken into account. The dataset was split into 50 sequence groups; each contained the corresponding guide sequence (gi|55981664) and the reference to the structure (1FNM) for threading. Computed alignments were coupled into one alignment and guide sequences were removed. This alignment was used for computing the phylogenetic tree of EFG subfamilies. Alignments for computing the phylogeny of EFG I, EFG II and for determining indels were computed by MAFFT using strategy L-INS-I [58].

### Estimating conserved positions

The EFG alignment was modified by removing all insertions relative to *Thermus thermophilus* EFG I (gi|55981664). Sequence logos for EFG subgroup alignments were calculated using the Sequence Logo website (version 2.8) [61]. The EFG I subfamily contained 114 sequences and the EFG II subfamily contained 140 sequences. These 114 sequences of EFG I are representing adequately conservation/variation pattern specific to the EFG I subfamily and incorporating more sequences from genomes with a single EFG gene does not change our results. The position was counted as conserved if the height of the sequence logo was at least three bits.

### Estimating position specific amino acid substitution rates

The relative site-specific substitution rates for EFG subfamilies were calculated by using Rate4Site [46] and ConSurf web server (http://consurf.tau.ac.il/) [47]. Alignments were computed by MAFFT using strategy L-INS-I [58]. More than 97% identical sequences were removed from the EFG I dataset resulted with 190 sequences (EFG I from all used genomes) and the EFG II subfamily contained 140 sequences. The run was carried out using PDB code 1FNM and the surface plot was generated using the PyMol script output by ConSurf [47].

### Methods used to predict fate of recent duplicates

Synonymous and non-synonymous substitution rate ratio was estimated by using codon models of sequence evolution implemented in CodeML [62]. Values of d_S_ (substitutions per synonymous site) and d_N_ (substitutions per non-synonymous site) were calculated as a cumulative value for the pair of sequences by using PAL2NAL [29]. When the ratio of d_N_/d_S_ (ω) is much lower than one (ω≪1) the gene is considered to be under selection, when close to one (d_N_/d_S_∼1) gene is considered to evolve under neutral model (no selection). Mutations accumulation is considered to be close to saturation when d_S_>3 and these pairs were removed from future analysis. To produce the figure of d_N_ as the function of d_S_ (Figure S3) equations (4) and (5) with predetermined values of free parameters [28] and the statistical software R [63] was used. Data points of d_N_ and d_S_, determined for recent duplicates of EFG I genes, were added to the figure. To determine the gene duplication event(s) on species tree for recent duplicates in β- and γ-proteobacteria a reconciliation tree between gene tree (EFG I) and species tree was computed by SoftParsMap [30]. 16S rRNA based species tree and EFG I protein sequence based tree were used as input for SoftParsMap [30]. Two alternative scenarios of gene gain and loss were mapped into improved species tree (Figure S4).

### Determining the type I and type II conserved positions

Positions that are highly conserved in the EFG I subfamily but where a different conserved amino acid in EFG II were identified (type I conserved positions). A preliminary set of such positions was obtained using the conservation criterion (3 bit). Only those positions where conservation of the different amino acid exceeds 80% in both subfamilies were selected. Positions that are conserved in EFG II but are relaxed in EFG I (type II conserved positions) were identified. In addition to the position conservation criterion (3 bit), the criterion for amino acid conservation (80%) in EFG II was utilized. In addition, the difference in amino acid conservation between subfamilies must exceed 25%.

### Computing phylogenetic trees

Bayesian tree searching was carried out using MrBayes 3.12 [64], [65] and a mixture of amino acid substitution models. Maximum likelihood trees were calculated with RAxML-VI-HPC 2.2.3 [66] using the PROTCATWAG amino acid substitution model. A gamma distribution with the α shape parameter estimated by the programs was used. Tree manipulations (computing consensus tree from RAxML bootstraps, joining groups in the tree and other simple manipulations) were carried out with MEGA3 [67].

For computing species trees, pre-aligned 16S rRNA sequences were downloaded from RDP II [68]. Bayesian tree searching was carried out with MrBayes 3.12 [64] under model GTR+I+Γ for up to 1 million iterations. For 214 EFG protein sequences (excluding *Leptospira interrogans* second EFGs) from 99 genomes (first dataset) Bayesian tree searching applied 2.5 million iterations.

To calculate the tree for EFG I, the second dataset was used. For rooting, one *Pirellula* EFG (gi|32475048 belonging to spdEFG2) was added. The multiple sequence alignment was generated with MAFFT version 5.861 [58] using strategy L-INS-i. Bayesian tree searching was applied up to 2.14 million iterations. For the subfamily of EFG II (third dataset), Bayesian tree searching was applied 5 million iterations and a maximum likelihood tree was calculated and bootstrapped 500 times.

### Finding genome context conservation

To determine the genome context of EFG genes, the orthologs of *E. coli* genes in other genomes were determined by INPARANOID [69]. For clustering genes with a similar set of neighboring genes, five genes before and after the gene of interest (EFG gene) were taken into account (not considering gene order). The distances between queried genes (EFG genes) were calculated on the basis of the number of common surrounding genes. A distance matrix was calculated in format, which served as input for the program NEIGHBOUR from the PHYLIP package [70]. This approach was useful for determining EFGs in the *str* operon. In other cases, the calculated similarity was manually rechecked as the capacity of the method to find similar genes is restricted to the gene repertory of *E. coli*.

## Supporting Information

Figure S1 **Alignment of EFG II-specific sub-subgroup consensus sequences.** EFG II sequences were aligned using mafft L-INSI and sub-subgroup-specific consensus was generated by python script cf.py, kindly supplied by Gemma Atkinson. To illustrate spdEFG1-specific three amino acid insertion into switch I, the first 80 positions of spdEFG1 consensus alignment are also shown. Conservation of positions is shown by: capitals (>70%), small letters (60%–70%), and dashes (<60%). Names of phyla/class specific sub-subgroups are shown on the left. The domain structure is shown as colored boxes: domains I, II, III, IV, and V are colored green (with the G′ olive green), blue, turquoise, brick red and orange respectively. Motifs of the GTPase domain (G-1 to G-5) are marked with light yellow boxes on alignment. P-loop, switch I and switch II are shown as light brown boxes. Indel regions are shown as rosy (insertion) and light blue (deletion) boxes. Indels are designated by Roman numerals from I to XI.
(TIF)

Figure S2 **Phylogenetic tree of EFG I type EFGs.** Tree was inferred using Bayesian inference (MrBayes v. 2.12). Tree contains EFGs originated from 303 genomes (first dataset). Formally, we distinguish between two sets of EFG I genes: first, EFGs from genomes with the single gene for EFG and second, EFGs from genomes with multiple genes for EFG. Names of the first set contain gi numbers and shortened name of the species. Names of the second set contain gi number, information on gene location (STR in str operon and nSTR outside str operon) and designation of phyla/class (see legends on the figure). Phyla/class borders are marked with gray/color lines at the right side of the figure. Colors on tree refer to recent duplications (green) and LGT (blue). Plausible duplication events are marked with a red arrow (also, look figure S3). Among genomes with a single EFG we found two cases where the corresponding gene was not found in the STR operon and these cases are marked with a red hexagon. Branches with higher posterior probability support than 0.5 are shown above branches. Scale bar corresponds to 0.5 changes per position. spdEFG from Pirellula sp. was used as an out-group.
(TIF)

Figure S3 **Substitutions pre non-synonymous site (dN) as a function of substitutions per silent site (dS).** Solid lines: middle line Eq. (4) (Hughes et al. 2007), lowest and highest lines are 5% and 95% quantiles of the distribution of dN for a given value of dS derived using Eq. (4) and (5) (Hughes et al. 2007). Dashed line: neutral model (dN = dS). Dot and dash line: sub-functionalization model (dN/dS = θ1). Red open circles: data points of β-proteobacteria recent duplicates (EFG I duplications).
(TIF)

Figure S4 **Two alternative scenarios of gene gain and loss for recent duplicates (EFG I duplications) presented in the clade tree of γ- and β-proteobacteria.** Arrows are indicating duplications and blue crosses deletions. Species names, where recent duplication was detected are colored red. The improved clade tree was produced from 16S rRNA species tree and EFG tree by using softparsmap.
(TIF)

Figure S5 **Checking incongruence between the EFG II phylogenetic tree and 16S rRNA-based species trees.** The EFG II tree was built using ML and BI methods as indicated in the materials and methods and Figure 3. EFG II specific sub-subgroups are indicated on the left side of figure. A species tree for each sub-subgroup was calculated using neighbor-joining method of MEGA3 and reliability was estimated by bootstrapping (500 times for each set). Only those branches that contain at least four sequences and in which branching order was reliably determined on both trees (shown as gray ovals) were used to determine plausible LGT events. Gray dotted lines connect reliably inferred branches of different trees, which contain sequences originating from the same species/genomes. Red dotted lines are indicate sequences that are displaced on one tree compared with another and therefore indicate to plausible events of LGT. All reliably detected LGTs stay inside the sub-subgroup.
(TIF)

Figure S6 **EFG domain conservation comparison for two major sub-subgroups of EFG II.** Column are colored: navy for EFG I; and red for EFG II. For calculating percentage of conserved positions for sub-subgroup of Clostridia (a) 25 sequences and for sub-subgroup alpha-proteobacteria/Cyanobacteria (b) 30 sequences were used. The domain conservation was estimated using sequence logos.
(TIF)

Figure S7 **Aligned sequence logos of EFG I and II.** Sequence logos were generated from alignment of 114 sequences of EFG I and 140 sequences of EFG II. All gaps were deleted according to EFG I (EF-G-1) from T. thermophilus. The bars above logos are colored by domain: domains I, II, III, IV, and V are colored green (with the G′ olive green), blue, cyan, ruby and orange respectively. Conserved motifs of GTPase domain are in gray boxes from G-1 to G-5. P-loop, switch I and II are in brown lines. Type I conserved positions are shown in yellow boxes and type II conserved positions in green boxes. Numeration in boxes corresponds to the EFG II structure 1WDT (EF-G-2 of T. thermophilus) and below the alignment EFG I (EF-G-1 T. thermophilus). RRF binding sites according to Gao N et al 2007 are shown in pink.
(TIF)
