# Supplementary figures and images for: A Computational Study of Elongation Factor G (EFG) Duplicated Genes: Diverged Nature Underlying the Innovation on the Same Structural Template
Source: PLoS One. 2011 Aug 4;6(8):e22789. doi: 10.1371/journal.pone.0022789 (PMC3150367; doi:10.1371/journal.pone.0022789)

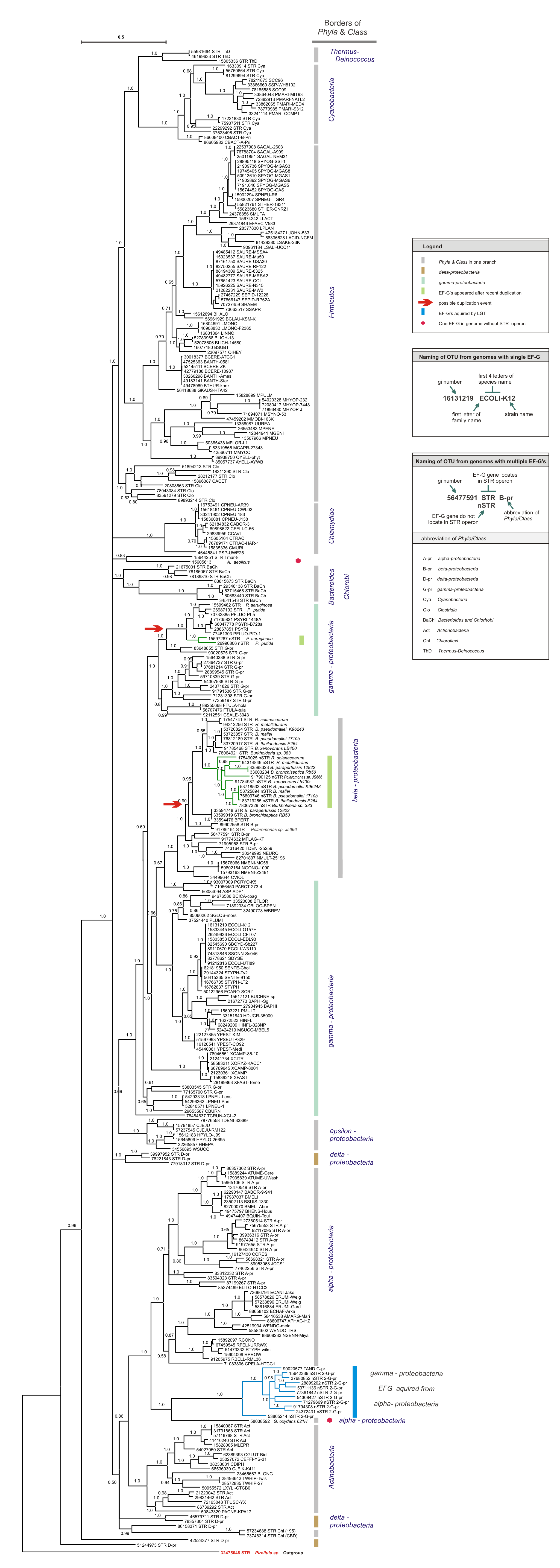

Supplement: Figure S2 — Phylogenetic tree of EFG I type EFGs. Tree was inferred using Bayesian inference (MrBayes v. 2.12). Tree contains EFGs originated from 303 genomes (first dataset). Formally, we distinguish between two sets of EFG I genes: first, EFGs from genomes with the single gene for EFG and second, EFGs from genomes with multiple genes for EFG. Names of the first set contain gi numbers and shortened name of the species. Names of the second set contain gi number, information on gene location (STR in str operon and nSTR outside str operon) and designation of phyla/class (see legends on the figure). Phyla/class borders are marked with gray/color lines at the right side of the figure. Colors on tree refer to recent duplications (green) and LGT (blue). Plausible duplication events are marked with a red arrow (also, look figure S3). Among genomes with a single EFG we found two cases where the corresponding gene was not found in the STR operon and these cases are marked with a red hexagon. Branches with higher posterior probability support than 0.5 are shown above branches. Scale bar corresponds to 0.5 changes per position. spdEFG from Pirellula sp. was used as an out-group. (TIF) [file pone.0022789.s002.tif]

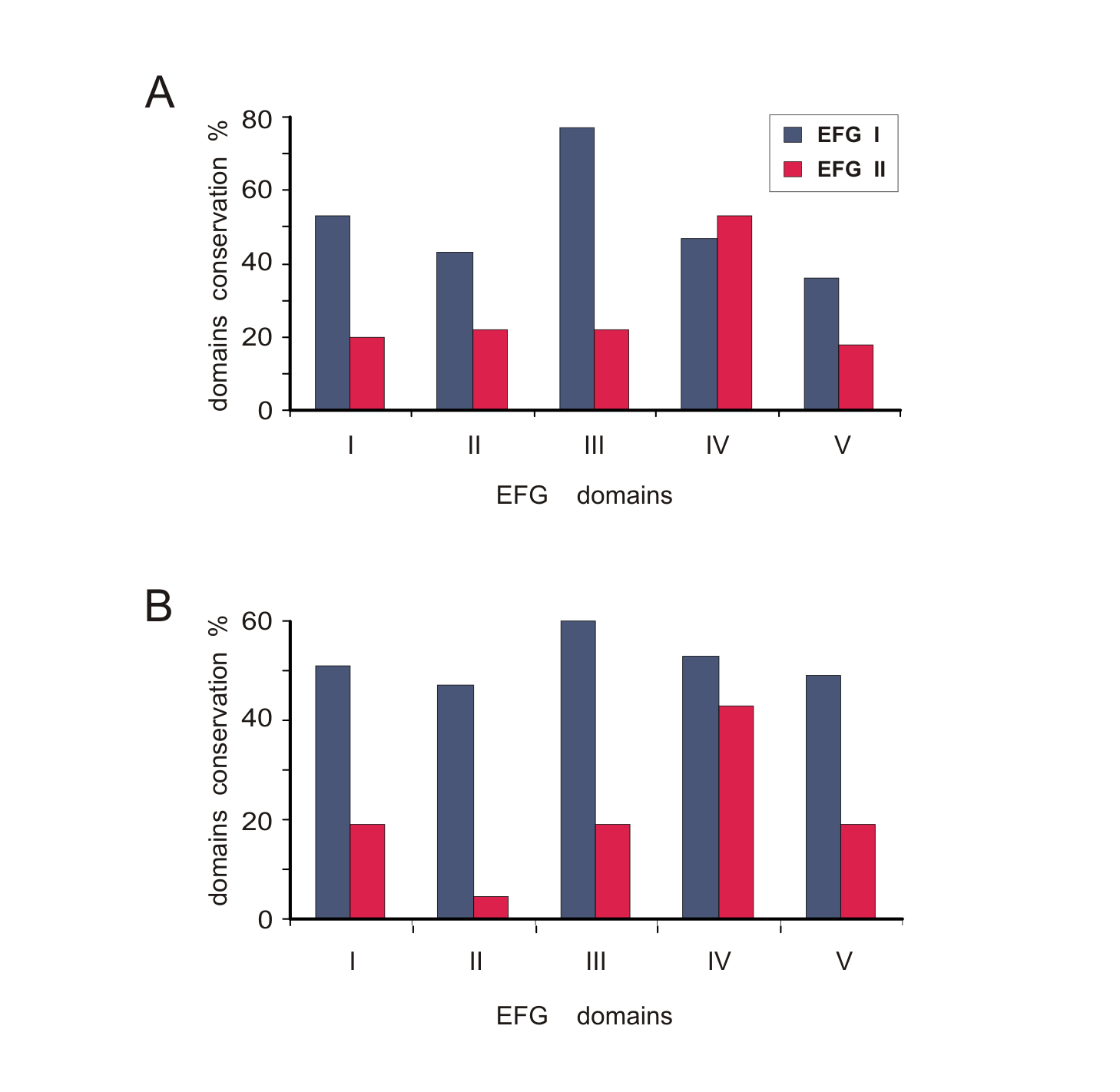

Supplement: Figure S6 — EFG domain conservation comparison for two major sub-subgroups of EFG II. Column are colored: navy for EFG I; and red for EFG II. For calculating percentage of conserved positions for sub-subgroup of Clostridia (a) 25 sequences and for sub-subgroup alpha-proteobacteria/Cyanobacteria (b) 30 sequences were used. The domain conservation was estimated using sequence logos. (TIF) [file pone.0022789.s006.tif]
